# On Disease of the Prostate

**Published:** 1881-09

**Authors:** 


### EXCERPTA.

On Disease of the Prostate.—At a recent meeting of the Harveian Society of London, Mr. Teevan read a paper on interference with micturition, the result of prostatic disease. It might be caused by, 1, acute inflammation; 2, chronic inflammation; 3, various alterations in bulk of the lobes of the organ; 4, stone descending into, or originating in the prostate; 5, malignant disease. All the above might, in some way or other, modify the act of micturition. Acute inflammation might be caused by the extension of gonorrhœal trouble, powerful injections, or tubercular and pyæmic deposits. If the patient could not micturate, an india rubber catheter ought to be at once introduced, as it was the instrument which caused the least irritation, but it was not always possible to pass it in such cases, and if it failed, a soft olivary catheter was indicated. By clearing out the rectum, filling it with ice, and placing half a dozen leeches on the perineum, the inflammation could usually be reduced, and the further employment of the catheter rendered unnecessary. If abscess formed, it generally burst into the urethra, but if fluctuation could be detected in the rectum or perineum, the knife ought to be at once applied. Chronic inflammation might follow gonorrhœa, or be caused by rectal trouble. The dull aching pain in the perineum and anus, associated with increased frequency of urination, was very wearying to a patient. The application of a few drops of a solution of sulphate of copper (five to ten grains to the ounce) to the prostatic urethra twice a week, would usually relieve the symptoms. If it failed, repeated blisterings of the perineum would generally effect a cure. Alterations in size of the different lobes of the prostate. It was of the utmost importance to detect prostatic obstruction early, for if it were not attended to, the bladder would become hypertrôphied and fasciculated, and the patient condemned to a life of misery. The earliest symptoms of commencing trouble were nocturnal frequency of micturition and the tendency of the urine to fall vertically between the patient's legs instead of being ejected in the arc of a circle. The sufferer might be taught to relieve his bladder night and morning with a soft olivary catheter. It could not be too strongly borne in mind that dribbling of urine in middle-aged and old men meant retention, not incontinence. If the practitioner were armed with a bougie catheter it was not necessary, in cases of retention from prostatic obstruction, to diagnose which particular lobe of the organ was enlarged, for the bougie would mount the hypertrophied median lobe, or would pass around the projecting lateral lobe. The catheter could then be screwed on to the bougie and made to follow the latter into the bladder, and so relieve the patient. A stone descending from the bladder might be arrested in the prostate, or have originated there. It was very important to make an accurate diagnosis, for cases have come under his notice which had been treated for retention from stricture where the obstruction was a stone in the prostate. If a metal instrument were not used the mistake might easily occur, for the sensation transmitted by the passage of a soft catheter through a urethra narrowed by a calculus in it, was almost similar to that conveyed by the instrument when introduced through a tight stricture. When a man who, up to the time of the stoppage, had always been able to pass a full stream of urine, was seized with retention, without any premonitory symptoms, the impaction of a stone in the prostatic urethra ought to be suspected. When a calculus was arrested in the deeper portion of the urethra it ought to be pushed back into the bladder with a wax bougie, and then crushed. Malignant disease of the prostate was, fortunately, very rare. It might not be attended by any pain, and could usually be detected by rectal examination, disclosing the existence of a hard nodule on one side of the prostate, and not on the other. This want of symmetry was a most valuable diagnostic sign. The few cases which had come under his notice were all of the scirrhous kind.—*Med. and Surg. Reporter.*

